# Relative Influence of Genetics and Shared Environment on Child Mental Health Symptoms Depends on Comorbidity

**DOI:** 10.1371/journal.pone.0103080

**Published:** 2014-07-31

**Authors:** Matthew K. Vendlinski, Kristin N. Javaras, Carol A. Van Hulle, Kathryn Lemery-Chalfant, Rose Maier, Richard J. Davidson, H. Hill Goldsmith

**Affiliations:** 1 Department of Psychology and Waisman Center, University of Wisconsin–Madison, Madison, Wisconsin, United States of America; 2 Department of Psychology, Arizona State University, Tempe, Arizona, United States of America; 3 Department of Psychology, University of Oregon, Eugene, Oregon, United States of America; University of Hong Kong, Hong Kong

## Abstract

**Background:**

Comorbidity among childhood mental health symptoms is common in clinical and community samples and should be accounted for when investigating etiology. We therefore aimed to uncover latent classes of mental health symptoms in middle childhood in a community sample, and to determine the latent genetic and environmental influences on those classes.

**Methods:**

The sample comprised representative cohorts of twins. A questionnaire-based assessment of mental health symptoms was used in latent class analyses. Data on 3223 twins (1578 boys and 1645 girls) with a mean age of 7.5 years were analyzed. The sample was predominantly non-Hispanic Caucasian (92.1%).

**Results:**

Latent class models delineated groups of children according to symptom profiles–not necessarily clinical groups but groups representing the general population, most with scores in the normative range. The best-fitting models suggested 9 classes for both girls and boys. Eight of the classes were very similar across sexes; these classes ranged from a “Low Symptom” class to a “Moderately Internalizing & Severely Externalizing” class. In addition, a “Moderately Anxious” class was identified for girls but not boys, and a “Severely Impulsive & Inattentive” class was identified for boys but not girls. Sex-combined analyses implicated moderate genetic influences for all classes. Shared environmental influences were moderate for the “Low Symptom” and “Moderately Internalizing & Severely Externalizing” classes, and small to zero for other classes.

**Conclusions:**

We conclude that symptom classes are largely similar across sexes in middle childhood. Heritability was moderate for all classes, but shared environment played a greater role for classes in which no one type of symptom predominated.

## Introduction

Patterns of family resemblance for psychopathology shed important light on its etiology. Although research on family resemblance for child and adolescent psychopathology has lagged behind that for adults, relevant findings regarding the former have recently begun to come into focus. For instance, Burt’s (2009) [1] recent meta-analysis of etiological influences on child and adolescent psychopathology allows for some broad generalizations. This meta-analysis included both twin and adoption studies of the etiology of conduct problems, oppositional defiant problems, attention-deficit/hyperactivity disorder (ADHD) symptoms, depression, and anxiety. Across all of these domains of psychopathology, genetic influences account for the largest proportion of variance, followed by unique (to each co-twin) environmental influences, and then shared (by both co-twins) environmental influences, although the limited role for shared environment may be partly an artifact of methodology [2], [3]. Notably, the contribution of genetic factors is slightly higher for externalizing problems (i.e., conduct problems, oppositional defiant problems, ADHD symptoms) than internalizing problems (i.e., depression and anxiety), whereas the contribution of non-shared environmental factors is slightly higher for internalizing problems than externalizing problems. However, one factor that limits these generalizations is the lack of consideration for comorbidity of symptoms from different domains.

Comorbidity between different types of mental health problems is common in childhood and adolescence [4]–[6]. One meta-analysis examining the comorbidity of depression, overanxiousness, conduct disorder (CD), and ADHD in childhood and adolescence found that every possible two-way combination of these disorders occurred at a rate well above chance [4]. These results derive from population-based samples; rates of comorbidity are typically even higher in clinical samples [7].

Despite the ubiquity of comorbidity, most studies of the latent genetic and environmental influences on childhood mental health symptoms have ignored comorbidity and focused on only one disorder or symptom scale. A few studies have included two disorders or symptom scales [8], [9]. Most relevant to our focus here, several studies have examined latent genetic and environmental influences on broadband internalizing/externalizing factors underlying multiple disorders [10]–[12]. These studies, which examine common (to all disorders or factors) and specific (to one disorder or factor) latent genetic and environmental influences, suggest that the relative contributions of these influences differ depending on whether symptoms/disorders occur in isolation or in combination. For example, these studies found that shared environment was implicated in common but not specific influences on internalizing and externalizing problems in five-year-old twins [11] and in five- to nine-year-old twins [10], although the role of shared environment in comorbid problems may diminish by adolescence [9], [10], [13]. Similarly, studies of measured genetic [14] and measured environmental [15] influences also suggest that results depend on the presence of comorbidity.

In contrast to the internalizing/externalizing factor approach to addressing comorbidity between multiple symptom domains, latent class analysis (LCA) is a person-centered approach that can be used to identify individuals with similar symptom profiles across multiple symptom domains [16]. Since LCA profiles differ in elevation and shape, LCA makes it possible to investigate etiological influences on different levels, and different combinations, of symptoms. Previous studies using LCA to characterize covariation among common mental health problems uncovered latent classes differentiated by the type and the severity of symptoms [17]–[19]. When symptoms of depression, anxiety, CD, oppositional defiant disorder (ODD), and ADHD are considered, six to eight latent classes are typically identified, and individual classes rarely map directly onto a single DSM-IV [20] label. For example, one study that used LCA to characterize covariation of ADHD, ODD, CD, and anxiety symptoms identified six common symptom profiles among 4–11 year olds [17]. These symptom profiles included a group of children with very few symptoms; a group with mild symptoms of inattention, ODD, CD, and anxiety; a group with moderate symptoms of inattention, impulsivity, ODD, CD, and anxiety; a group with moderate symptoms of impulsivity, ODD, and CD; a group with severe symptoms of inattention, impulsivity, ODD, CD, and anxiety; and a group with severe symptoms of inattention, impulsivity, ODD, and CD. Although some LCA studies have used twins and have included a range of symptoms (e.g., [18]), few attempted to estimate the heritability of class membership,. Our study moves this body of research forward by characterizing etiological influences on both symptom type and symptom level through genetic/environmental analyses of latent class results. Further, our approach affords the opportunity to estimate genetic and environmental contributions to the absence of psychopathology, as indicated by low symptom levels across multiple symptom domains.

Our aims were twofold. First, we used LCA to characterize typical symptom profiles in middle childhood using symptoms of depression, separation anxiety, overanxiousness, conduct disorder, oppositional defiant disorder, and ADHD. Our aim was *not* to suggest an alternative to current or proposed diagnostic nosology; rather, we sought to examine symptom patterns in the broader population and whether these patterns appear to be similar across boys and girls. Second, we estimated the extent to which latent genetic and environmental influences contributed as sources of variation in these patterns, with equal emphasis on normative and more clinical patterns, the latter of which describe fewer children.

## Methods

### Participants and Procedure

Data came from a birth-record-based sample of twins born in the state of Wisconsin beginning in 1989 [21], [22]. Of families with infant twins, approximately 65% of those we attempted to contact, whether contact was ever made or not, agreed to participate. As twins approached seven years of age, we attempted to recruit all families for the middle childhood phase of data collection, with funding being the only constraint on recruitment (i.e., we did not select twins for follow-up based on their characteristics). Approximately 78% of the families agreed to participate, and caregivers were administered questionnaires over the telephone assessing demographic characteristics, twin zygosity, and child health and behavior. Child health and behavior were assessed using the MacArthur Health and Behavior Questionnaire (HBQ) [23], [24], which includes questions assessing DSM-defined symptoms. Parents of the children provided written informed consent following appropriate ethical guidelines, and the protocol was approved by the Social and Behavioral Sciences IRB at the University of Wisconsin–Madison.

We performed latent class analyses on child behavior items (i.e., DSM-defined symptoms) from the HBQ. Concurrent and predictive validity for the LCA results was provided by additional measures (referred to as ‘external covariates’) administered during (‘concurrent’) and shortly after (‘subsequent’) the middle childhood phase. The concurrent external covariates include demographic information and information on impairment from the HBQ. The subsequent external covariates include diagnoses from a diagnostic interview administered to mothers during a follow-up to the middle childhood assessment; the follow-up occurred when children were 8 years old on average (SD = .87 years). By design, 40% of the middle childhood sample participated in the follow-up.

We included 3223 twins (boys = 1578; girls = 1645) with mostly complete HBQ data in latent class analyses because we wanted the classes to be based on as broad a sample of the population as possible. In the LCA sample, the mean age was 7.5 years (SD = .92), and 92.1% of the children were non-Hispanic Caucasian. (Primary caregivers classified the race and ethnicity of children using options provided by study investigators.) Before performing the external covariate and genetic/environmental analyses for the resulting latent classes, we removed 35 boys and 12 girls with developmental disorder diagnoses (primarily autism) or major medical problems (e.g., spina bifida) because behavioral problems in these participants might have a different etiology than in the rest of the sample. Thus, the external covariate analyses included 1543 male and 1633 female twins, although demographics were available for approximately 85–90% and follow-up diagnoses were available only for approximately 40% (mostly by design, as described above). In the genetic/environmental analyses, we excluded twins whose zygosity was unclear, as well as twins who did not have a cotwin who could be included (i.e., due to missing data, developmental disorder, or major medical problem). The resulting sample for genetic/environmental analyses included 256 monozygotic (MZ) and 267 dizygotic (DZ) male twin pairs, 318 MZ and 240 DZ female twin pairs, and 479 opposite-sex DZ twin pairs.

### Measures

#### Zygosity

Zygosity was established via the 32-item Zygosity Questionnaire for Young Twins [25], which measures aspects of physical similarity, confusion of twins, and medical information (e.g., chorionicity), and which yields >95% agreement with genotyping [26]. A follow-up with other zygosity determination procedures, including genotyping, was used for a portion of the twins.

#### Socioeconomic Status

We assessed socioeconomic status (SES) using caregiver report of maternal and paternal educational attainment and gross annual family income. On average, mothers had 14.9 years (SD = 2.3) of education whereas fathers had 14.6 (SD = 2.6) years. The distribution of annual family incomes was as follows: 4.5% below $20,000, 6.5% between $20,001 and $30,000, 23.7% between $30,001 and $50,000, 23.5% between $50,001 and $70,000, 17.4% between $70,001 and $90,000, and 24.4% above $90,001.

#### Mental Health Problems (Middle Childhood Assessment)

Primary caregivers (approximately 98% mothers and 2% fathers) reported child mental health symptoms and associated functional impairment exhibited during the previous six months via the HBQ. Caregivers rated whether statements describing DSM-based symptoms were never, somewhat, or often true of their children. Items measuring symptoms of depression (6 items), overanxiousness (12 items), separation anxiety (10 items), conduct problems (12 items), oppositional defiant problems (9 items), impulsivity (9 items), and inattention (6 items) were included in LCA. Caregivers also provided information on the degree to which children were impaired by their symptoms via the Impairment on Self scale (8 items; e.g., “How much trouble has your child had getting along with his/her teacher(s) as a result of the behaviors or behavior problems you identified in the previous section?”) and the Impairment on Family scale (8 items); the mean composite scores for these scales were used as external covariates.

#### Mental Health Problems (Follow-Up to Middle Childhood Assessment)

Approximately 6 to 9 months after the middle childhood assessment, DSM-IV diagnoses were obtained for children via caregiver report on the Diagnostic Interview Schedule for Children Version IV (DISC-IV) [27]. Staff administered this fully structured interview during a visit to the family home. We used past year diagnoses generated from the major depressive disorder, generalized anxiety disorder, separation anxiety disorder, conduct disorder, oppositional defiant disorder, and ADHD modules.

### Statistical Analysis

We performed LCA, followed by external covariate analyses and genetic/environmental analyses for the best-fitting latent classes. We chose to use LCA–rather than factor mixture models that allow a within-class severity dimension and thus have less restrictive assumptions (e.g., [28], [29])–to characterize symptom patterns for conceptual reasons (e.g., patterns with more clinical elevations may not represent merely the extremes of more temperament-based patterns [30]). We conducted LCA separately for boys and girls because previous studies identified important sex differences in the structure of mental health problems [18], [31]. Indeed, our own analyses yielded some differences across sexes. We also performed the external covariate analyses separately for boys and girls. However, genetic/environmental analyses were performed after combining boys and girls with similar symptom profiles to increase power. We did not perform genetic/environmental analyses for latent classes that were found for girls only or boys only.

LCA is a type of latent variable modeling that assumes each individual belongs to one of K latent classes, in contrast to latent trait or factor analysis models, which assume that individuals fall along one or more latent continua. In LCA, the manifest variables are also categorical (e.g., ordinal categorical in the case of the HBQ items), and the manifest variables are assumed to be independent within a given class (i.e., assumption of local independence) [32]. The two types of parameters for each latent class are class membership probability, which describes the proportion of individuals falling into that latent class, and item-class parameters characterizing average probabilities of endorsement for each HBQ item for individuals in that latent class. These latter parameters determine the item response (or symptom) profile for the class. Although latent classes in LCA are not assumed to be ordered, finding that the estimated item response profiles for two or more classes have similar patterns differing only in level of endorsement (i.e., severity) suggests those classes are ordered.

To estimate the parameters for each LCA model, we used LatentGOLD software [33], which employs an Expectation Maximization (EM) algorithm [34] to maximize the likelihood for the model. To ensure the EM algorithm found the global maximum of the likelihood for each LCA model, we specified that LatentGold use 1000 sets of random starting values for each fit, and we fitted each model 5 times to ensure that the resulting values of the maximized likelihood were identical. For each sex, we fitted latent class models with K = 1 through K = 11 latent classes. To decide which model fit best for each sex, we selected the model with the lowest Bayesian Information Criterion (BIC) [35]. We also used the bootstrapping function in LatentGOLD to ensure the best-fitting model fit well according to a bootstrapped p-value for the chi-square test because the usual (i.e., non-bootstrapped) p-value may not be valid for data (such as ours) where some combinations of item responses are not observed. Finally, we repeated the procedure just described, but with a family-specific random effect included in each LCA model to account for dependence between cotwins because doing so can give different results regarding the number of classes [36]. For our data, however, BIC results for boys and for girls followed a similar pattern with and without a family-specific random effect. Thus, in all subsequent analyses, we used results from the best-fitting LCA models without a family-specific random effect.

We used item-class parameters for the best-fitting LCA models to plot and describe the item response profiles for each class, for girls and for boys. We describe each class at two levels of detail. To decide on more detailed names, we considered patterns of symptom elevation relative to other classes, in addition to absolute levels. In deciding on less detailed names, which were intended to capture only the most distinctive features of the item response profile for that class, we based names in part on the 10 items with highest endorsement (relative to the “Low Symptom” group) for the class in question. We use the less-detailed names to refer to the classes throughout the text for the sake of brevity.

For the external covariate analyses, we used the probabilities of class membership from the best-fitting LCA models to calculate a weighted average of the external covariates for participants in each class. For each external covariate, we informally compared the various class-specific averages to investigate whether the external covariate varied across the symptom-defined classes as would be expected. For example, we would expect to find the highest levels of self-impairment and family impairment in the moderate and severe symptom classes.

For the genetic/environmental analyses, we used two approaches. In the first approach, we calculated odds ratios (the odds of being in latent class k given that your co-twin is in latent class j divided by the odds of being in latent class k given that your co-twin is not in latent class j) separately for twins of each zygosity, using either the probabilities of class membership for each participant or the modal class assignment (based on the largest probability of class membership for each participant). To calculate the odds ratios and test their significance, we used logistic regression with sex included as a covariate.

In our second approach, we used modal class assignments to fit biometric twin models [37] for membership in each class (but not for cross-class membership). For each class, we used a liability-threshold approach that assumes an unobserved continuous liability for membership in a given class, with membership in the class occurring when the liability exceeds a sex-specific threshold corresponding to the prevalence of the class for the relevant sex. To ensure the identification of the model parameters, the liability distribution was assumed to be standard normal [38]. Variation in this liability was decomposed into underlying genetic (A), shared environmental (C), and non-shared environmental (E) variation. Models were fit directly to raw data using the structural equation program Mx [39]. To assess fit of the ACE model for a given latent class, we compared the loglikelihood for the ACE model to the loglikelihood for a saturated model. Under certain conditions, –2(LL_ACE_–2LL_SATURATED_) follows a chi-square distribution with 2 degrees of freedom.

## Results

### LCA Results

**Figure 1:**
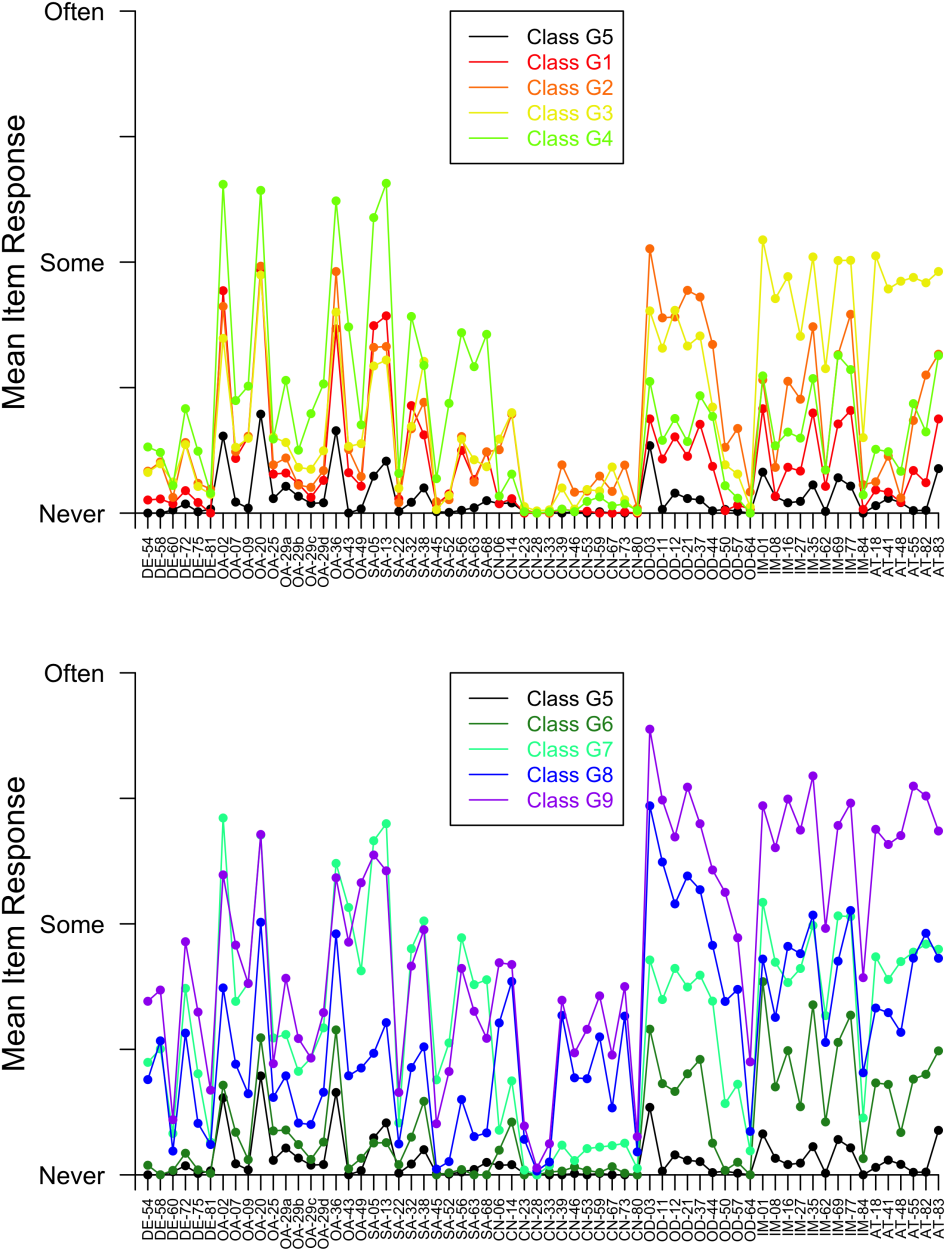
Item response plots for girls’ latent classes G5 and G1–G4 (upper panel) and girls’ latent classes G5 and G6–G9 (lower panel). Abbreviations: DE = Depression; OA = Overanxiousness; SA = Separation Anxiety; CN = Conduct Problems; OD = Oppositional Defiant Problems; IM = Impulsivity; AT = Inattention.

**Figure 2:**
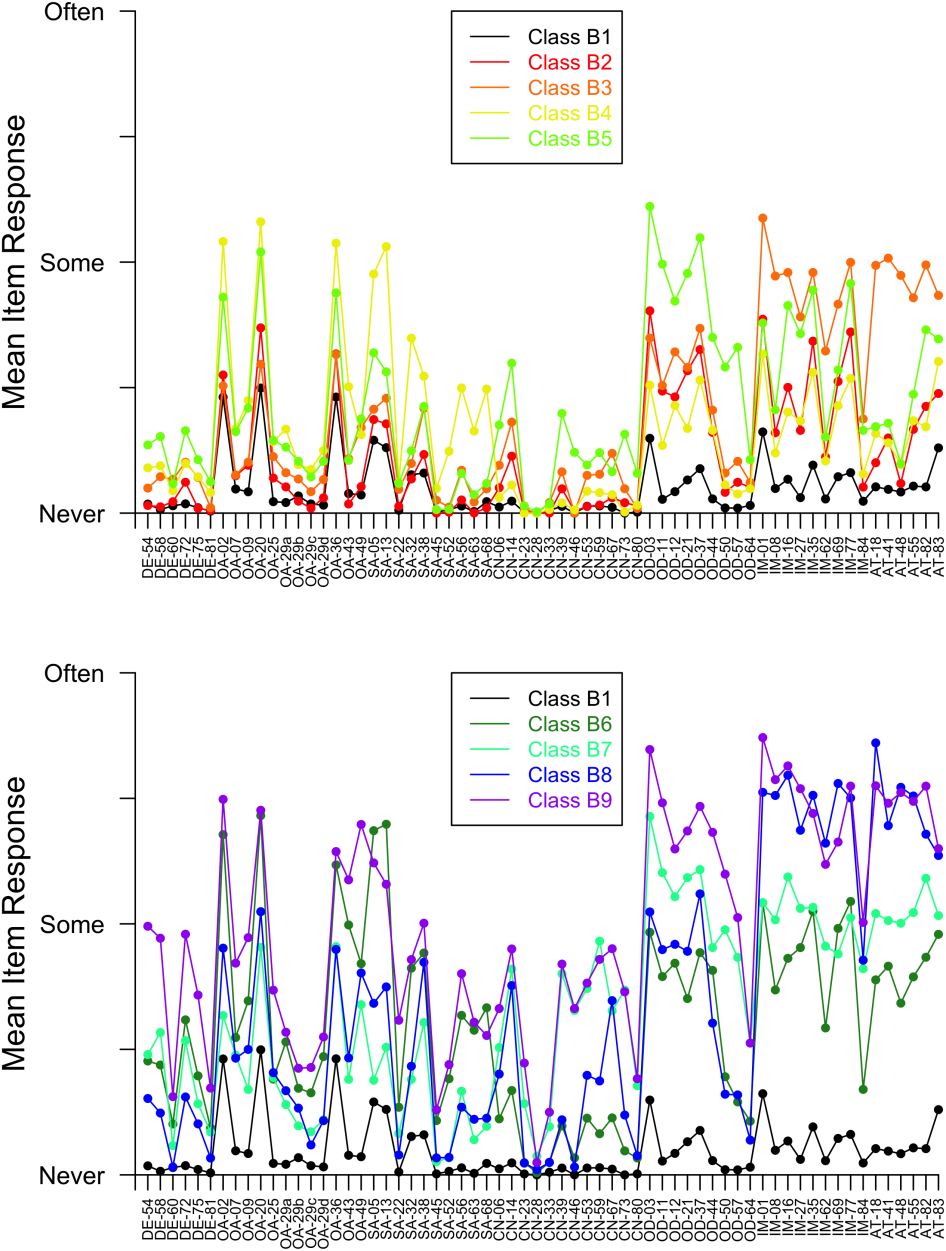
Item response plots for boys’ latent classes B1 and B2–B5 (upper panel) and boys’ latent classes B1 and B6–B9 (lower panel). Abbreviations: DE = Depression; OA = Overanxiousness; SA = Separation Anxiety; CN = Conduct Problems; OD = Oppositional Defiant Problems; IM = Impulsivity; AT = Inattention.

For girls, BIC was smallest for the 9-class model; further, the bootstrapped p-value from the chi-square test of goodness of fit was 0.35 for the 9-class model, suggesting that this model fit the girls’ data well. For boys, BIC was smallest for the 9-class and 10-class models (BIC values for these models were very similar), but we chose the 9-class model for comparability with girls. The bootstrapped p-value from the chi-square test of goodness of fit was 0.33 for the 9-class model, suggesting this model fit the boys’ data well. Class names and membership probabilities are presented in Table 1, and symptom profile plots are presented in Figures 1a and 1b for girls and Figures 2a and 2b for boys.

**Table 1:** Class descriptions and probabilities of class membership for best-fitting latent class models.

Class	Brief Class Description	Detailed Class Description	Probability of ClassMembership
Girls			
G1	Mildly Anxious	Mildly Anxious, Mildly Oppositional, Mildly Impulsive	0.16
G2	Moderately Oppositional	Mildly Anxious, Mildly Impulsive, Moderately Oppositional	0.15
G3	Moderately Impulsive &Inattentive	Mildly Anxious, Moderately Oppositional, Moderately Impulsive,	0.14
		Moderately Inattentive	
G4	Moderately Anxious	Moderately Anxious, Mildly Oppositional, Mildly Impulsive, MildlyInattentive	0.12
G5	Low Symptom	Low Symptom	0.11
G6	Mildly Oppositional & Impulsive	Mildly Oppositional, Mildly Impulsive, Mildly Inattentive	0.11
G7	Moderately Anxious & non-Conduct	Mildly Depressed, Moderately Anxious, Moderately Oppositional,	0.09
	Externalizing	Moderately Impulsive, Moderately Inattentive	
G8	Moderately Externalizing	Mildly Depressed, Mildly Anxious, Moderate Conduct Problems,	0.08
		Severely Oppositional, Moderately Impulsive, Moderately Inattentive	
G9	Moderately Internalizing & Severely	Moderately Depressed, Moderately Anxious,	0.04
	Externalizing	Moderate Conduct Problems, Severely Oppositional, SeverelyImpulsive,	
		Severely Inattentive	
Boys			
B1	Low Symptom	Low Symptom	0.17
B2	Mildly Oppositional & Impulsive	Mildly Oppositional, Mildly Impulsive	0.17
B3	Moderately Impulsive & Inattentive	Mildly Oppositional, Moderately Impulsive, Moderately Inattentive	0.13
B4	Mildly Anxious	Mildly Anxious, Mildly Oppositional, Mildly Impulsive	0.13
B5	Moderately Oppositional	Mildly Anxious, Moderately Oppositional, Moderately Impulsive	0.11
B6	Moderately Anxious & non-Conduct	Moderately Anxious, Moderately Oppositional, ModeratelyImpulsive,	0.10
	Externalizing	Moderately Inattentive	
B7	Moderately Externalizing	Mildly Depressed, Mildly Anxious, Moderate Conduct Problems,	0.08
		Severely Oppositional, Moderately Impulsive, ModeratelyInattentive	
B8	Severely Impulsive & Inattentive	Mildly Depressed, Mildly Anxious, Mild Conduct Problems,	0.06
		Moderately Oppositional, Severely Impulsive, Severely Inattentive	
B9	Moderately Internalizing & Severely	Moderately Depressed, Moderately Anxious, Moderate ConductProblems,	0.05
	Externalizing	Severely Oppositional, Severely Impulsive, Severely Inattentive	

The following 8 classes had remarkably similar profiles across sexes. A “Low Symptom” class, designated G5 for the girls and B1 for the boys in Figures 1a through 2b, showed little elevation across symptoms. Interestingly, this class had the highest probability of class membership for boys (0.17), but the 5^th^ highest for girls (0.11). Two mildly elevated profiles were identified: a “Mildly Anxious” class with slight elevations on overanxiousness and separation anxiety items, and a “Mildly Oppositional & Impulsive” class. Four moderately elevated profiles emerged: a “Moderately Oppositional” class; a “Moderately Impulsive & Inattentive” class; a “Moderately Anxious & non-Conduct Externalizing” class with elevations on overanxiousness, separation anxiety, oppositional, impulsivity, and inattention items; and a “Moderately Externalizing” class with elevations on conduct, oppositional, impulsivity, and inattention items. Finally, a “Moderately Internalizing & Severely Externalizing” class was identified for both sexes. This class showed moderate elevations across depression, overanxiousness, separation anxiety, and conduct items along with more extreme elevations on oppositional, impulsivity, and inattention items. In addition to these 8 classes, a “Moderately Anxious” class was identified in girls but not boys, and a “Severely Impulsive & Inattentive” class was found for boys but not girls. Next, we turn to analyses that expand upon the meaning of these classes.

### External Covariates

Class-specific averages of parental education, family income, impairment, and DISC-IV diagnoses are presented in Table 2 (girls) and Table 3 (boys). These averages follow expected patterns, providing external support for the validity of the LCA results. With regard to concurrent validity, self and family impairment scores are highest in the two classes (“Moderately Externalizing” and “Moderately Internalizing & Severely Externalizing”) most elevated on externalizing items for both sexes. Similarly, parental education and family income are lowest in theses same two most symptomatic class for both sexes, particularly for classes G9 and B9.

**Table 2:** Weighted^a^ averages of external covariates for girls, by latent class.

		Demographics (Concurrent)			Impairment (Concurrent)		Diagnoses (Subsequent)						
		Maternal Education(yrs)	PaternalEducation(yrs)	Family IncomeCategory^b^	Self Impair-Ment^c^	Family Impair-Ment^c^	MDD (%)	GAD (%)	SAD (%)	CD (%)	ODD (%)	ADHD (%)	Any Diag-nosis^d^ (%)
G1	Mildly Anxious	14.9	14.9	10.7	0.05	0.05	0.9	0.0	2.9	0.0	3.0	0.0	6.8
G2	Moderately Oppositional	15.1	14.8	10.9	0.17	0.14	0.2	0.0	0.3	2.6	4.7	0.3	8.0
G3	Moderately Impulsive & Inattentive	14.9	14.4	10.4	0.17	0.17	0.0	0.0	2.2	0.1	3.3	16.6	22.1
G4	Moderately Anxious	15.0	14.9	10.4	0.15	0.13	0.0	3.1	9.3	0.0	1.4	0.1	12.3
G5	Low Symptom	14.8	14.8	9.8	0.02	0.01	0.0	0.0	1.0	0.0	1.0	0.0	2.1
G6	Mildly Oppositional & Impulsive	15.2	15.1	10.7	0.07	0.08	1.1	1.1	0.0	0.0	2.4	2.3	4.7
G7	Moderately Anxious & non-Conduct Externalizing	14.3	14.4	10	0.30	0.26	2.4	1.5	20.5	1.5	4.4	6.0	30.4
G8	Moderately Externalizing	15.0	14.7	10.2	0.32	0.31	0.0	0.0	4.7	10.0	19.4	3.9	30.1
G9	Moderately Internalizing & Severely Externalizing	14.2	13.7	8.3	0.59	0.78	3.1	4.5	13.9	11.9	39.2	20.2	62.8

a Weighted by class membership probabilities.

b Income categories: 8 = $40,001 to $45,000; 9 = $45,001 to $50,000; 10 = $50,001 to $60,000; 11 = $60,001 to $70,000; 12 = $70,001 to $80,000.

c Self Impairment Scale ranges from 0 (no impairment) to 1.9 (highest observed impairment) and Family Impairment Scale ranges from 0 (no impairment) to 2.8 (highest observed impairment).

d Any diagnosis refers to a diagnosis of MDD, GAD, SAD, CD, ODD, or ADHD.

Abbreviations: MDD = Major Depressive Disorder; GAD = Generalized Anxiety Disorder; SAD = Separation Anxiety Disorder; CD = Conduct Disorder; ODD = Oppositional Defiant Disorder; ADHD = Attention-Deficit/Hyperactivity Disorder.

**Table 3:** Weighted^a^ averages of external covariates for boys, by latent class.

		Demographics (Concurrent)			Impairment (Concurrent)		Diagnoses (Subsequent)						
		Maternal Education(yrs)	Paternal Education(yrs)	Family IncomeCategory^b^	Self Impair-ment^c^	Family Impair-ment^c^	MDD (%)	GAD (%)	SAD (%)	CD (%)	ODD (%)	ADHD (%)	Any Diag-nosis^d^ (%)
B1	Low Symptom	15.2	15.2	11.0	0.03	0.04	0.0	0.0	0.7	0.6	2.4	1.8	4.9
B2	Mildly Oppositional& Impulsive	15.3	14.9	10.6	0.09	0.10	0.0	0.0	0.1	1.8	3.0	9.0	13.1
B3	Moderately Impulsive &Inattentive	14.9	14.4	10.3	0.19	0.22	0.0	0.0	0.7	3.1	3.6	10.3	16.6
B4	Mildly Anxious	15.0	14.4	10.8	0.11	0.12	0.0	0.0	9.3	3.3	3.7	0.2	16.4
B5	ModeratelyOppositional	15.2	14.7	10.8	0.25	0.25	0.0	0.0	1.5	5.8	18.1	8.9	28.6
B6	Moderately Anxious& non-ConductExternalizing	15.0	14.6	10.2	0.25	0.26	0.0	1.6	13.8	4.6	9.8	16.5	35.4
B7	ModeratelyExternalizing	14.6	14.0	10.1	0.43	0.52	0.1	0.0	1.8	16	31.4	21.9	49.4
B8	Severely Impulsive &Inattentive	14.6	14.1	10.8	0.31	0.34	3.9	0.0	3.9	5.0	12.7	25.8	39.8
B9	Moderately Internalizing &SeverelyExternalizing	14.1	13.4	8.4	0.73	0.98	1.9	0.0	11.6	29.3	35.1	21.4	64.3

a Weighted by class membership probabilities.

b Income categories: 8 = $40,001 to $45,000; 9 = $45,001 to $50,000; 10 = $50,001 to $60,000; 11 = $60,001 to $70,000; 12 = $70,001 to $80,000.

c Self Impairment scale ranges from 0 (no impairment) to 1.9 (highest observed impairment) and Family Impairment Scale ranges from 0 (no impairment) to 3.0 (highest observed impairment).

d Any diagnosis refers to a diagnosis of MDD, GAD, SAD, CD, ODD, or ADHD.

Abbreviations: MDD = Major Depressive Disorder; GAD = Generalized Anxiety Disorder; SAD = Separation Anxiety Disorder;

CD = Conduct Disorder; ODD = Oppositional Defiant Disorder; ADHD = Attention-Deficit/Hyperactivity Disorder.

With regard to predictive validity, the proportion of children subsequently diagnosed with conduct disorder was highest in the most symptomatic class. For boys, the three classes with the highest levels of impulsivity and inattention (“Moderately Externalizing,” “Severely Impulsive & Inattentive,” and “Moderately Internalizing & Severely Externalizing”) predict the highest rates of subsequent ADHD diagnoses. For girls, the three classes with the largest elevations on overanxiousness and separation anxiety items (“Moderately Anxious,” “Moderately Anxious & non-Conduct Externalizing,” and “Moderately Internalizing & Severely Externalizing”) are at greatest risk for subsequent separation anxiety diagnoses. Thus, membership in more symptomatic latent classes (based on the HBQ) predicts later diagnoses of a similar nature (based on the DISC-IV).

### Genetic/Environmental Analyses

The monozygotic and dizygotic twin odds ratios based on class probabilities (see Table S1 and Table S2) were very similar to those based on modal class assignment (see Table S3 and Table S4). Their similarity suggests fitting biometric twin models to modal class assignments will produce valid estimates of additive genetic (A), shared, or common, environmental (C), and non-shared, or unique, environmental (E) variance, even though modal class assignments ignore some of the information contained in the class probabilities.

Estimates and corresponding confidence intervals for A, C, and E from the biometric twin models are presented in Table 4. The estimates of genetic variance are moderate (approximately 0.30 to 0.50) for all classes. In addition, the estimates of shared environmental variance are moderate for the “Low Symptom” and “Moderately Internalizing & Severely Externalizing” classes, small for the “Moderately Anxious & non-Conduct Externalizing” and “Moderately Externalizing” classes, and zero for the other classes. The width of the confidence intervals suggests that we did not have sufficient power to test whether the variance components differed from zero in some cases, especially for the less prevalent classes.

**Table 4:** Proportion of variance accounted for (confidence intervals) and model fit of biometric twin models for girls and boys combined.

Girls and Boys Combined	A^a^	C^a^	E^a^		*–2LL*	*–2LL*	*Δχ^2^*
					*Sat*	*ACE*	
C1. Mildly Anxious	0.42 (0.07–0.53)	0.0 (0.0–0.25)	0.58 (0.44–0.73)		2578.4	2559.1	19.3***
C2. Moderately Oppositional	0.47 (0.29–0.60)	0.0 (0.0–0.11)	0.53 (0.40–0.68)		2397.2	2377.8	19.4***
C3. Moderately Impulsive & Inattentive	0.51 (0.34–0.64)	0.0 (0.0–0.9)	0.49 (0.36–0.64)		2428.8	2424.7	4.1
C5. Low Symptom	0.29 (0.0–0.54)	0.49 (0.26–0.69)	0.26 (0.16–0.36)		2371.6	2332.7	38.9***
C6. Mildly Oppositional & Impulsive	0.44 (0.02–0.57)	0.01 (0.0–0.33)	0.55 (0.42–0.71)		2446.3	2434.1	12.2**
C7. Moderately Anxious & non-Conduct Externalizing	0.33 (0.0–0.69)	0.22 (0.0–0.53)	0.45 (0.29–0.62)		1881.0	1867.2	13.8**
C8. Moderately Externalizing	0.37 (0.0–0.70)	0.17 (0.0–0.50)	0.46 (0.30–0.65)		1750.8	1732.8	18.0***
C9. Moderately Internalizing & Severely Externalizing	0.40 (0.0–0.79)	0.30 (0.0–0.74)	0.29 (0.16–0.56)		1109.5	1095.5	14.0**

a A, C, and E refer to proportion of variance accounted for by additive genetic, shared environment and unique environment, respectively.

Abbreviations: Sat = saturated model; ACE = twin model; LL = log-likelihood; Δχ***^2^*** = delta chi square;

*p<.05,

**p<.01,

***p<.001.

## Discussion

Latent class analyses of common mental health symptoms measured in middle childhood revealed nine distinct profiles of problems for boys and for girls. These profiles reflect symptomatology only, unlike classifications based on DSM-IV diagnoses, which require that symptoms be accompanied by clinically significant impairment. Eight of the profiles were very similar for both sexes. A “Low Symptom” class with almost no problems emerged for both sexes. For the remaining classes, all items were endorsed more frequently than in the “Low Symptom” class, but certain items had more extreme elevations. Two mildly elevated classes were identified: a mildly anxious class and a mildly disruptive class. Four moderately elevated classes were identified: three that encompassed largely disruptive symptoms and one that encompassed both anxious and disruptive symptoms. The mildly elevated classes might be viewed as temperamental variation rather than symptom-based variation in the population, and this temperamental interpretation might partially apply to the moderately elevated classes as well.

The most severe class encompassed comorbid internalizing and externalizing symptoms. The external covariate analyses showed that this was the only class where impairment was substantially elevated and where the majority of children subsequently qualified for DSM-IV diagnoses. This lack of symptom differentiation among the more severely impaired children may be a function of our derivation of classes from a community sample, but may also highlight the notion of a general disposition to more serious but common psychopathology, sometimes called the p-factor [40].

Finally, the two sex-specific classes were a “Moderately Anxious” class in girls only and a “Severely Impulsive & Inattentive” class in boys only. This latter class makes sense, given the sex differences in prevalence of ADHD [5]. Although comparing class prevalence across sexes is not possible because the latent class analyses were performed separately by sex, our pattern of results suggests that the types of problem behavior classes found in boys and girls in middle childhood are generally very similar.

Latent class analyses can inform our understanding of the structure of child mental health symptoms. For instance, the profiles that emerged from our analyses shed light on whether oppositional, impulsive, and inattentive symptoms represent one (oppositional + impulsive + inattentive), two distinct (oppositional + impulsive vs. inattentive), or three distinct (oppositional vs. impulsive vs. inattentive) dimensions of symptoms [31]. Although our approach was not set up to directly test the relative fit of these competing models, the covariation of oppositional, impulsive, and inattentive items across the different latent classes provides important information. In our best-fitting solution, the various class profiles differed in shape for those items. In many classes (e.g., “Moderately Impulsive & Inattentive” and “Moderately Anxious & non-Conduct Externalizing”), the oppositional, impulsivity, and inattention items are similarly elevated. However, in some classes (e.g., “Moderately Oppositional”), the oppositional and impulsivity items are considerably more elevated than the inattention items, and in the “Severely Impulsive & Inattentive” class for boys, the impulsivity and inattention items were more elevated than the oppositional items. These detailed results suggest that oppositional, impulsive, and inattentive symptoms do not reflect a single dimension. In particular, impulsive behaviors can occur alongside oppositional problems, attentional problems, or both, possibly reflecting distinct psychological and biological underlying processes.

Biometric modeling of the genetic and environmental influences on the latent classes for boys and girls combined suggests that genetic variation plays a moderate role in all classes, including the “Low Symptom” class representing the absence of symptoms of psychopathology, which is a novel finding. Another novel finding is that shared environment appears to play a moderate role in only the “Low Symptom” and “Moderately Internalizing & Severely Externalizing” classes, and at most a small role in the other classes. These findings suggest that shared environment plays a larger role in the etiology of profiles with similar elevations (or similar lack of elevations) for internalizing and externalizing symptoms (i.e., profiles with similar levels of comorbid internalizing and externalizing symptoms). Our results are consistent with previous findings of moderate shared environmental correlations between anxiety and conduct problems in two- to four-year-old twins [8] and between internalizing and externalizing symptoms in four- to eleven-year-old twins [12]. Our results are also consistent with previous studies where shared environment was implicated in common but not specific influences on internalizing and externalizing problems in five-year-old twins [11] and in five- to nine-year-old twins [10]. Although these previous studies and ours suggest that shared environmental factors play a moderate role in the etiology of comorbid internalizing and externalizing problems in early and middle childhood, the role of shared environment in comorbid problems may diminish by adolescence [9], [10], [13].

Our latent class approach allows us to examine genetic and environmental influences on not only mental health symptoms, but also on the lack thereof (i.e., the “Low Symptom” class). Although genetic influences may play a role in membership in the “Low Symptom” class, shared environmental influences play a moderate and statistically significant role. These shared environmental influences may represent the absence of certain familial risk factors for psychopathology (e.g., poverty, domestic violence), but may also represent the presence of certain protective familial factors (e.g., social support).

One limitation of our study is that the latent class analyses were based on mental health symptoms reported via questionnaire by only one informant (the primary caregiver). Thus, we cannot rule out the possibility that the “Low Symptom” classes included children whose primary caregivers were not willing to acknowledge any problem behaviors for either twin, which would lead to inflated estimates of shared environmental influences. However, in analyses using a subset of the LCA sample that had mother, father, and child reports available (results not reported), the latent classes identified and the pattern of genetic and environmental influences on those classes were very similar across informants.

Another potential limitation is our use of a community-based sample. Given that the majority of our sample would not qualify for a psychiatric diagnosis, it is uncertain how well our results would generalize to clinical populations. However, a recent factor analytic study of adults suggests the structure of mental health problems is similar across community and clinical samples [41]. Further, using a community-based sample also increases the representativeness of results and eliminates treatment-seeking biases.

In conclusion, our study identified nine symptom profiles present during middle childhood. Eight profiles are similar across sexes, but a “Moderately Anxious” class only appeared in girls and a “Severely Impulsive & Inattentive” class was found only in boys. We emphasize that these symptoms profiles are not proposed as superior to existing nosology. Rather, they elucidate the patterning of symptoms across the severity range in a general population, with some classes that are virtually free of clinically significant psychopathology. Although genetic influences play a moderate role in the development of all classes, shared environment plays a moderate role in comorbid internalizing and externalizing problems and in the absence of either type of problem. Our findings suggest that accounting for covariation across symptom domains can reveal the impact of shared environment on the development of common mental health problems. The symptom profiles characterized in this study demonstrate that comorbidity is common in childhood and provide clinicians with common patterns to be aware of as they assess children.

## Supporting Information

Table S1 **Combined-sex probability-based odds ratios (p-values) for monozygotic twins.**
(DOCX)

Table S2 **Combined-sex probability-based odds ratios (p-values) for dizygotic twins.**
(DOCX)

Table S3 **Combined-sex modal-assignment-based odds ratios (p-values) for monozygotic twins.**
(DOCX)

Table S4 **Combined-sex modal-assignment-based odds ratios (p-values) for dizygotic twins.**
(DOCX)
