# Supplementary material for: Relative Influence of Genetics and Shared Environment on Child Mental Health Symptoms Depends on Comorbidity
Source: PLoS One. 2014 Jul 31;9(7):e103080. doi: 10.1371/journal.pone.0103080 (PMC4117501; doi:10.1371/journal.pone.0103080)
Supplement: Table S1 — Combined-sex probability-based odds ratios (p-values) for monozygotic twins. (DOCX) [file pone.0103080.s001.docx]

**Table S1. Combined-sex probability-based odds ratios^a^ (p-values) for monozygotic twins**

|  |  | **Twin 2** | | | | | | | |
| --- | --- | --- | --- | --- | --- | --- | --- | --- | --- |
|  |  | C1. | C2. | C3. | C5. | C6. | C7. | C8. | C9. |
| **Twin 1** | C1. Mildly Anxious | 5.557 (0.000) | 0.261 (0.018) | 0.244  (0.014) | 2.192  (0.009) | 1.947  (0.034) | 0.040  (0.020) | 0.015  (0.058) | 0.146  (0.127) |
|  | C2. Moderately Oppositional | 1.269 (0.522) | 10.000 (0.000) | 0.459  (0.122) | 0.067  (0.001) | 0.992  (0.982) | 0.721  (0.537) | 1.316  (0.583) | 0.101  (0.150) |
|  | C3. Moderately Impulsive & Inattentive | 0.192  (0.011) | 0.606  (0.287) | 9.890  (0.000) | 0.286  (0.010) | 0.875  (0.715) | 1.371  (0.471) | 0.731  (0.591) | 0.622  (0.563) |
|  | C5. Low Symptom | 0.129  (0.031) | 0.100  (0.033) | 0.006  (0.088) | 53.312  (0.000) | 0.736  (0.465) | 0.000  (0.304) | 0.000  (0.336) | 0.000  (0.525) |
|  | C6. Mildly Oppositional & Impulsive | 0.807  (0.634) | 0.522  (0.201) | 0.488  (0.145) | 1.852  (0.052) | 6.439  (0.000) | 0.000  (0.150) | 0.140  (0.054) | 0.176  (0.198) |
|  | C7. Moderately Anxious &  non-Conduct Externalizing | 0.663  (0.417) | 1.126  (0.791) | 1.301  (0.532) | 0.008  (0.041) | 0.146  (0.012) | 9.145 (0.000) | 0.638  (0.520) | 1.857  (0.346) |
|  | C8. Moderately Externalizing | 0.354  (0.100) | 1.482  (0.366) | 1.276  (0.582) | 0.000  (0.055) | 0.215  (0.026) | 1.029  (0.959) | 10.539  (0.000) | 4.612  (0.006) |
|  | C9. Moderately Internalizing & Severely Externalizing | 0.317  (0.312) | 0.237  (0.293) | 0.511  (0.488) | 0.002  (0.418) | 0.177  (0.176) | 4.547  (0.009) | 5.197  (0.006) | 17.681  (0.000) |

^a^ Odds ratios (the odds of twin 2 being in class k given that twin 1 was in class j / the odds of twin 2 being in class k given that twin 1 was not in

class j) were calculated using logistic regression with sex included as a covariate.
