# Supplementary material for: Relative Influence of Genetics and Shared Environment on Child Mental Health Symptoms Depends on Comorbidity
Source: PLoS One. 2014 Jul 31;9(7):e103080. doi: 10.1371/journal.pone.0103080 (PMC4117501; doi:10.1371/journal.pone.0103080)
Supplement: Table S2 — Combined-sex probability-based odds ratios (p-values) for dizygotic twins. (DOCX) [file pone.0103080.s002.docx]

**Table S2. Combined-sex probability-based odds ratios^a^ (p-values) for dizygotic twins**

|  |  | **Twin 2** | | | | | | | |
| --- | --- | --- | --- | --- | --- | --- | --- | --- | --- |
|  |  | C1. | C2. | C3. | C5. | C6. | C7. | C8. | C9. |
| **Twin 1** | C1. Mildly Anxious | 2.064  (0.004) | 0.845  (0.604) | 0.944  (0.858) | 0.937  (0.812) | 1.160  (0.627) | 0.608  (0.246) | 0.362  (0.051) | 0.654  (0.385) |
|  | C2. Moderately Oppositional | 1.027  (0.924) | 1.211  (0.510) | 1.457  (0.192) | 0.493  (0.028) | 0.495  (0.068) | 2.253  (0.008) | 0.709  (0.417) | 0.858  (0.735) |
|  | C3. Moderately Impulsive & Inattentive | 0.858  (0.581) | 1.771  (0.025) | 1.291  (0.361) | 0.692  (0.194) | 1.358  (0.278) | 0.497  (0.101) | 1.199  (0.604) | 0.487  (0.161) |
|  | C5. Low Symptom | 0.400  (0.024) | 0.350  (0.024) | 0.434  (0.061) | 12.361  (0.000) | 1.217  (0.539) | 0.165  (0.022) | 0.273  (0.048) | 0.094  (0.046) |
|  | C6. Mildly Oppositional & Impulsive | 1.078  (0.783) | 0.841  (0.579) | 0.550  (0.101) | 2.467  (0.000) | 2.897  (0.000) | 0.167  (0.007) | 0.424  (0.079) | 0.200  (0.028) |
|  | C7. Moderately Anxious &  non-Conduct Externalizing | 0.803  (0.513) | 0.900  (0.765) | 0.874  (0.712) | 0.080  (0.000) | 0.233  (0.012) | 5.295  (0.000) | 1.797  (0.103) | 2.228  (0.035) |
|  | C8. Moderately Externalizing | 1.361  (0.316) | 1.075  (0.834) | 1.206  (0.591) | 0.089  (0.001) | 0.437  (0.089) | 0.663  (0.405) | 4.203  (0.000) | 1.731  (0.197) |
|  | C9. Moderately Internalizing & Severely Externalizing | 0.723  (0.512) | 0.995  (0.992) | 0.498  (0.274) | 0.126  (0.023) | 0.286  (0.116) | 0.920  (0.888) | 2.627  (0.026) | 8.569  (0.000) |

^a^ Odds ratios (the odds of twin 2 being in class k given that twin 1 was in class j / the odds of twin 2 being in class k given that twin 1 was not in

class j) were calculated using logistic regression with sex included as a covariate.
