# Supplementary material for: Relative Influence of Genetics and Shared Environment on Child Mental Health Symptoms Depends on Comorbidity
Source: PLoS One. 2014 Jul 31;9(7):e103080. doi: 10.1371/journal.pone.0103080 (PMC4117501; doi:10.1371/journal.pone.0103080)
Supplement: Table S3 — Combined-sex modal-assignment-based odds ratios (p-values) for monozygotic twins. (DOCX) [file pone.0103080.s003.docx]

**Table S3. Combined-sex modal-assignment-based odds ratios^a^ (p-values) for monozygotic twins**

|  |  | **Twin 2** | | | | | | | |
| --- | --- | --- | --- | --- | --- | --- | --- | --- | --- |
|  |  | C1. | C2. | C3. | C5. | C6. | C7. | C8. | C9. |
| **Twin 1** | C1. Mildly Anxious | 4.988  (0.000) | 0.239  (0.007) | 0.321  (0.018) | 1.739  (0.043) | 1.567  (0.118) | 0.099  (0.024) | 0.129  (0.046) | 0.246  (0.178) |
|  | C2. Moderately Oppositional | 1.614  (0.144) | 6.693  (0.000) | 0.377  (0.043) | 0.171  (0.001) | 0.771  (0.444) | 0.868  (0.759) | 1.180  (0.726) | 0.000  (0.989) |
|  | C3. Moderately Impulsive & Inattentive | 0.227  (0.015) | 0.632  (0.278) | 8.809  (0.000) | 0.344  (0.010) | 0.819  (0.557) | 1.131  (0.777) | 0.538  (0.317) | 0.682  (0.615) |
|  | C5. Low Symptom | 0.112  (0.032) | 0.112  (0.032) | 0.000  (0.985) | 25.241  (0.000) | 0.757  (0.470) | 0.000  (0.985) | 0.000  (0.985) | 0.000  (0.991) |
|  | C6. Mildly Oppositional & Impulsive | 0.690  (0.386) | 0.675  (0.357) | 0.640  (0.269) | 1.458  (0.187) | 3.885  (0.000) | 0.000  (0.982) | 0.147  (0.062) | 0.302  (0.251) |
|  | C7. Moderately Anxious &  non-Conduct Externalizing | 0.617  (0.374) | 1.251  (0.608) | 1.325  (0.496) | 0.070  (0.009) | 0.171  (0.016) | 11.155  (0.000) | 0.952  (0.937) | 2.001  (0.289) |
|  | C8. Moderately Externalizing | 0.542  (0.258) | 1.337  (0.483) | 1.266  (0.567) | 0.000  (0.977) | 0.265  (0.029) | 1.266  (0.639) | 10.667  (0.000) | 3.782  (0.016) |
|  | C9. Moderately Internalizing & Severely Externalizing | 0.360  (0.326) | 0.000  (0.986) | 0.353  (0.316) | 0.000  (0.978) | 0.246  (0.176) | 4.500  (0.007) | 4.331  (0.014) | 18.941  (0.000) |

^a^ Odds ratios (the odds of twin 2 being in class k given that twin 1 was in class j / the odds of twin 2 being in class k given that twin 1 was not in

class j) were calculated using logistic regression with sex included as a covariate.
