# Supplementary material for: Relative Influence of Genetics and Shared Environment on Child Mental Health Symptoms Depends on Comorbidity
Source: PLoS One. 2014 Jul 31;9(7):e103080. doi: 10.1371/journal.pone.0103080 (PMC4117501; doi:10.1371/journal.pone.0103080)
Supplement: Table S4 — Combined-sex modal-assignment-based odds ratios (p-values) for dizygotic twins. (DOCX) [file pone.0103080.s004.docx]

**Table S4. Combined-sex modal-assignment-based odds ratios^a^ (p-values) for dizygotic twins**

|  |  | **Twin 2** | | | | | | | |
| --- | --- | --- | --- | --- | --- | --- | --- | --- | --- |
|  |  | C1. | C2. | C3. | C5. | C6. | C7. | C8. | C9. |
| **Twin 1** | C1. Mildly Anxious | 1.878  (0.007) | 0.975  (0.930) | 1.247  (0.444) | 0.779  (0.320) | 1.119  (0.695) | 0.685  (0.330) | 0.391  (0.049) | 0.837  (0.672) |
|  | C2. Moderately Oppositional | 0.947  (0.841) | 1.216  (0.478) | 1.463  (0.171) | 0.523  (0.027) | 0.632  (0.188) | 2.484  (0.001) | 0.660  (0.313) | 1.088  (0.832) |
|  | C3. Moderately Impulsive & Inattentive | 0.848  (0.524) | 1.671  (0.034) | 1.303  (0.320) | 0.659  (0.104) | 1.459  (0.149) | 0.648  (0.240) | 1.054  (0.874) | 0.501  (0.150) |
|  | C5. Low Symptom | 0.336  (0.007) | 0.295  (0.010) | 0.424  (0.049) | 9.163  (0.000) | 1.155  (0.643) | 0.284  (0.036) | 0.296  (0.043) | 0.129  (0.043) |
|  | C6. Mildly Oppositional & Impulsive | 1.026  (0.919) | 0.868  (0.617) | 0.525  (0.064) | 2.220  (0.000) | 2.223  (0.001) | 0.199  (0.007) | 0.554  (0.150) | 0.191  (0.023) |
|  | C7. Moderately Anxious &  non-Conduct Externalizing | 0.991  (0.975) | 0.858  (0.654) | 1.261  (0.471) | 0.111  (0.000) | 0.300  (0.021) | 4.666  (0.000) | 1.585  (0.188) | 2.008  (0.060) |
|  | C8. Moderately Externalizing | 1.436  (0.204) | 1.265  (0.455) | 1.225  (0.542) | 0.132  (0.001) | 0.439  (0.083) | 0.624  (0.325) | 3.869  (0.000) | 1.430  (0.399) |
|  | C9. Moderately Internalizing & Severely Externalizing | 0.720  (0.501) | 0.923  (0.871) | 0.594  (0.394) | 0.091  (0.019) | 0.368  (0.173) | 0.848  (0.788) | 2.492  (0.037) | 9.310  (0.000) |

^a^ Odds ratios (the odds of twin 2 being in class k given that twin 1 was in class j / the odds of twin 2 being in class k given that twin 1 was not in

class j) were calculated using logistic regression with sex included as a covariate.
